# Commentary: Transcatheter aortic valve replacement in a quadricuspid aortic valve: a systematic review and meta-analysis

**DOI:** 10.3389/fcvm.2025.1660716

**Published:** 2026-01-27

**Authors:** Rajeh S. H. Ruzayqat

**Affiliations:** Faculty of Medicine, Hebron University, Hebron, Palestine

**Keywords:** aortic stenosis, QAV, quadricuspid aortic valves, systematic review, transcatheter aortic valve replacement

A Commentary on Transcatheter aortic valve replacement in a quadricuspid aortic valve: a systematic review and meta-analysis By Khalifa MA, Hashim HT, Shimal AA, Riyas Mohamed FR, Ragunathan S, Al Sakini AS, Elbadawi MH, Irfan MR, Moqbel I, Almualed MM, Al-Ghuraibawi M and Al-Aboudi BS (2025). Front. Cardiovasc. Med. 12: 1572251. doi: 10.3389/fcvm.2025.1572251.

Khalifa et al. (2025) conducted a systematic review evaluating the use of transcatheter aortic valve replacement (TAVR) in patients with a quadricuspid aortic valve (QAV). The study identified 11 case reports/series (*n* = 17) and summarized procedural characteristics and short-term outcomes (1).

The study title and the methods section state that a random-effects meta-analysis was performed, “A random-effects model was used for meta-analysis,” to assess procedural success and 30-day mortality. However, a detailed examination of the reported statistical methods and results does not identify a quantitative synthesis consistent with a meta-analysis. The analyses are conducted at the individual-patient level using a one-stage descriptive approach, as mentioned in the methods section, “Individual participants’ data analysis was used. The analysis approach was a one-stage approach,” without study-level effect estimates, variance calculations, measures of heterogeneity (e.g., *I*^2^), or graphical meta-analytic outputs such as forest or funnel plots.

Although the authors state that a random-effects model was used, the described analytical approach does not reflect a random-effects meta-analysis as conventionally defined. In the absence of pooled estimates derived from multiple studies, the methodology more closely aligns with a descriptive systematic review of case reports rather than a meta-analysis.

Referring to the study as a “meta-analysis” may therefore overstate the level of quantitative evidence provided and could mislead readers regarding the strength and generalizability of the findings. Clarification of the analytical framework or a reconsideration of the terminology used in the title and methods would improve methodological transparency.
